# Characteristics of rapid cycling in 1261 bipolar disorder patients

**DOI:** 10.1186/s40345-023-00300-z

**Published:** 2023-06-04

**Authors:** Alessandro Miola, Leonardo Tondo, Marco Pinna, Martina Contu, Ross J. Baldessarini

**Affiliations:** 1 Department of Psychiatry, University of Padova, Padua, Italy; 2 International Consortium for Mood & Psychotic Disorders Research, Mailman Research Center, McLean Hospital, Belmont, Massachusetts USA; 3 Lucio Bini Mood Disorder Centers, Cagliari & Rome, Italy; 4 Department of Psychiatry, Harvard Medical School, Boston, Massachusetts USA; 5 Section on Psychiatry, Department of Medical Science and Public Health, University of Cagliari, Cagliari, Italy

**Keywords:** Bipolar disorder, Episode frequency, Morbidity, Rapid-cycling course, Risk factors, Temperament

## Abstract

**Background:**

Rapid-cycling (RC; ≥ 4 episodes/year) in bipolar disorder (BD) has been recognized since the 1970s and associated with inferior treatment response. However, associations of single years of RC with overall cycling rate, long-term morbidity, and diagnostic subtypes are not clear.

**Results:**

We compared descriptive and clinical characteristics in 1261 BD patients with/without RC, based on history and prospective follow-up for several years. RC in any previous year was identified in 9.36% of BD subjects (3.74% in BD1, 15.2% BD2), and somewhat more among women than men. RC-BD subjects had 3.21-fold greater average prospective annual rates of recurrence but not hospitalizations, had less difference in %-time-ill, received more mood-stabilizing treatments, and had greater suicidal risk, lacked familial psychiatric illnesses, had more cyclothymic temperament, were more likely to be married, had more siblings and children, experienced early sexual abuse, but were *less* likely to abuse drugs (not alcohol) or smoke. In multivariable regression modeling, older age, mood-switching with antidepressants, and BD2 > BD1 diagnosis, as well as more episodes/year were independently associated with RC. Notably, prospective mean recurrence rates were *below* 4/year in 79.5% of previously RC patients, and below 2/year in 48.1%.

**Conclusions:**

Lifetime risk of RC in BD was 9.36%, more likely in women, with older age, and in BD2 > BD1. With RC, recurrence rates were much higher, especially for depression with less effect on %-time ill, suggesting shorter episodes. Variable associations with unfavorable outcomes and prospective recurrence rates well below 4/year in most previously RC patients indicate that RC was not a sustained characteristic and probably was associated with use of antidepressants.

## Background

The concept of "rapid cycling" (RC) was proposed nearly five decades ago by (Dunner and Fieve 1974) to describe bipolar manic-depressive patients with an inferior prophylactic response to lithium treatment and an episode recurrence rate of at least four/year. The criterion of four mood episodes to describe these difficult-to-treat patients was chosen arbitrarily to obtain sufficiently large samples to study (Dunner et al. 1977). Nevertheless, the validity of RC as a distinct course modifier for bipolar disorder (BD) was to some degree supported in subsequent investigations (Bauer et al. 1994; Maj et al. 1994]. RC is currently accepted as a specifying characteristic of BD, considered as the occurrence of at least four episodes of mania, hypomania, depression, or mixed-state during the previous 12 months in the APA Diagnostic and Statistical Manual of Mental Disorders (DSM) as well as the WHO International Classification of Diseases (ICD). Episodes are demarcated in DSM by partial or full remission for at least two months or a switch to an episode of opposite polarity (APA 2022). Lifetime prevalence of RC in BD patients (≥ 4 episodes within any 12-month period) broadly ranges from 10 to 43% (Fountoulakis et al. 2013; Carvalho et al. 2014), indicating probable variance among places and methods of sampling and case identification.

As expected, prevalence of the RC phenomenon was considerably higher when the critical defining criterion was ≥ 4 recurrences of discrete major affective illness episodes in any year (lifetime risk), with weighted means of 31.5%, but only 18.1% if the exposure criterion was limited to the previous year (Carvalho et al. 2014). Previous evidence syntheses revealed strong associations of RC-BD with childhood maltreatment, mixed manic-depressive features in recurrent episodes, more suicide attempts, and poor response to mood-stabilizers (Tondo et al. 2003; Agnew-Blais and Danese 2016; Dualibe and Osorio 2017; Dong et al. 2019; Hui et al. 2019; Bartoli et al. 2020; Grillault-Laroche et al. 2020), whereas suggestive associations with longer illness, predominant depressive polarity, and type II BD (BD2) diagnosis were supported by less extensive and consistent evidence (Kupka et al. 2003; Carvalho et al. 2014; Sentissi et al. 2019). Some reports found RC-BD to be more prevalent among BD2 patients (Kilzieh and Akiskal 1999; Erol et al. 2015), but others found an excess among those diagnosed with bipolar I disorder (BD1) (Schneck et al. 2004; Kupka et al. 2005). In addition, predominance of depression the long-term clinical course was noted in some reports (Coryell et al. 2003; Schneck et al. 2004; Calabrese et al. 2005), although predominance of hypo[mania] with RC-BD was found in another study and in association with higher episode frequency (Kupka et al. 2005).

Of particular importance, there has been little information regarding whether RC-BD is a sustained characteristic of BD patients or a manifestation of instability that waxes and wanes over time, as few reports have addressed the long-term morbidity in RC-BD patients. Based on following 109 RC-BD patients for 2–36 years, 33% achieved remission for at least a year, 40% continued being RC with severe episodes, 14% maintained RC but with less severe episodes, and 13% cycled at lower rates (Koukopoulos et al. 2003). In the Systematic Treatment Enhancement Program for Bipolar Disorder (STEP-BD) follow-up study, most (66%) of the 356 patients with prior RC had further recurrences but at the end of 12 months, 34% had no further mood episodes, another 34% experienced one episode, and 27% had two or three episodes, and only 5% of the patients had  ≥ 4 recurrences within 1 year (Schneck et al. 2008).

Earlier illness-onset and greater illness severity, particularly regarding depressive symptoms and level of functioning, have been reported as important clinical predictors of episode recurrence (Schneck et al. 2008; Valentí et al. 2015; Peters et al. 2016). Moreover, the likelihood of RC in BD patients is probably increased with antidepressant use (Koukopoulos et al. 2003; Schneck et al. 2008; El-Mallakh et al. 2015; Valentí et al. 2015). Despite the original characterization of RC-BD patients as poor responders to lithium treatment (Dunner et al. 1977), lithium has been at least as effective in RC patients as alternatives (Tondo et al. 2003), and optimal treatment of the condition remains uncertain. Additional features distinguishing between RC-BD patients who achieved remission from those who continued to cycle rapidly included a previous course-pattern of depression before [hypo] mania and then a euthymic interval (DMI) rather than the opposite (MDI), the occurrence of mood switching from depression to [hypo] mania during antidepressant treatment, and occurrence of agitated depression (Koukopoulos et al. 2003).

Nevertheless, the range of long-term courses in patients meeting criteria for RC-BD and the prognostic value of the RC construct remain poorly understood. The need to clarify questions remaining unresolved encouraged the present analyses of data arising from a large, extensively and repeatedly evaluated cohort of DSM-5-TR-based BD patients at mood disorder centers in Sardinia and Rome.

## Methods

### Subjects

This study involved a large cohort of consecutive adult participants evaluated and followed at specialized outpatient clinics for assessment and treatment of affective disorders: the Lucio Bini Mood Disorders Centers in Cagliari and Rome, Italy. Subjects were clinically diagnosed with a bipolar disorder (bipolar-I [BD1], or bipolar-II [BD2]), updated to meet DSM-5-TR criteria (APA 2022). They underwent systematic evaluation based on retrospective information at intake and repeated, prospective assessments during follow-up, with semi-structured interviews and individual life-charts in use since the 1970s—all by the same mood disorder expert (LT). Morbidity was quantified during follow-up assessments at the study sites for subjects followed for at least one year after intake and considered by historical review for earlier years. Clinical data were recorded systematically and converted to digitized form, with diagnoses of prospectively assessed morbid states updated to meet DSM-5-TR criteria. Participants provided written informed consent at clinic entry for potential anonymous reporting of clinical data in aggregate analyses, in accordance with requirements of Italian law (IMEF 2014).

### Clinical measures

We assessed information for comparison of bipolar disorder (BD) patients with versus without a rapid cycling course (RC), defined as having four or more discrete episodes of illness as major depression, episodes with mixed manic-depressive features, mania or hypomania (“[hypo] mania”) within *any* 12-month period between illness onset and clinic entry. In addition to basic demographic and descriptive data, we considered diagnosis (BD1 vs. BD2), sex, age, and clinical factors of interest. We also considered *psychometric features* at clinic intake, including affective temperament scored with the 39-item version of the self-assessment TEMPS-A scale (Akiskal et al. 2005a, b), depression severity using the 21-item Hamilton Depression Rating Scale (HDRS_21_) (Hamilton 1960), current [hypo]manic status with the Young Mania Rating Scale (YMRS) (Young et al. 1978), and anxiety with the Hamilton Anxiety Rating Scale (HARS) (Hamilton 1959), as well as functional status with the Global Assessment of Functioning (GAF) scale (Monrad-Aas 2011).

Illness course-related variables included: age at illness onset (as well as at first symptoms and first treatment), type of first lifetime episode, (depressive, manic or hypomanic, mixed, or psychotic), duration of the first-lifetime episode and of the first inter-episode interval. We also assessed the predominant long-term morbidity type (> 30% depressive or > 20% [hypo] manic), or predominant long-term course-sequencing (as Depression-[Hypo] mania-euthymic Interval [DMI] or [Hypo] mania-Depression-Interval [MDI]) with the support of life charting. Related measures of morbidity included the observed annual frequency of illness-episodes (all episodes, depressions, [hypo] manias) and the average percentage of time-at-risk in any morbidity or in depression or [hypo] mania, all based on retrospective information and progressive evaluations over at least 12 months of follow-up. We also addressed the distribution of individual mean recurrence rates (episodes/years at risk) during prospective follow-up. Retrospective morbidity indices were compared to those during follow-up to evaluate effects of clinical interventions at the study centers.

### Data analysis

Data are presented as means with 95% confidence intervals [CI]. Sociodemographic and selected clinical data were analyzed for differences between BD subjects with versus without rapid cycling (RC). Comparisons were based on contingency tables (χ^2^) for categorical measures and analysis of variance (*t*-test) for continuous measures. These subgroup comparisons were ranked by the significance of differences of measures between those with versus without RC. We also used multivariable logistic regression modeling to identify factors that were significantly and independently associated with RC status. The distribution of prospective recurrence rates was compared for RC versus nonRC subjects using cumulative histograms. Statistical significance was limited to guiding selection of initial measures for further analyses, but also generally considering two-tailed *p* ≤ 0.01 to indicate particularly interesting findings as well as to compensate for multiple comparisons. Analyses employed commercial software: *Statview.5* (SAS Institute, Cary, NC) for spreadsheets, and *Stata.1*7 (StataCorp, College Station, TX) for analyses.

## Results

### Risk of rapid cycling

This study involved 1261 DSM-5-TR bipolar disorder (BD) participants, 642 BD1 and 619 BD2, with 722 women and 539 men. Age at intake averaged 45.0 and 39.4 years among those with (n = 118) or without (n = 1143) previous rapid-cycling (RC) defined as ≥ 4 discrete, major DSM-5-TR mood episodes (mania or hypomania [“(hypo) mania”], major depression, or a state with mixed features) within *any* 12-month period (Table 1).

**Table 1:** Rapid cycling (RC) in bipolar disorder patients

Factors	Cases (n)	RC rate (%)	RR	χ^2^	*p*-value
All cases	1261	9.36 [7.81–11.1]	–	–	–
Bipolar type					
BD1	642	3.74 [2.41–5.51]	4.06	48.7	< 0.0001
BD2	619	15.2 [12.4–18.3]			
Sex					
Women	722	10.9 [8.76–13.4]	1.51	5.00	0.025
Men	539	7.24 [5.20–9.76]			
Diagnosis and sex			[*]		
BD2 Women	370	16.5 [12.9–20.7]	[comparator]		
BD1 Women	352	13.3 [9.30–18.1]	1.24		
BD2 Men	249	5.11 [3.06–7.96]	3.23		
BD1 Men	290	2.07 [0.76–4.49]	7.97	–	–

*RR* = rate ratio. [*] Compares prevalence to BD2 women as the comparison group

RC was identified in 118/1261 (9.36% [CI 7.81–11.1]) BD subjects overall), with four-times higher risk with BD2 (15.2% [12.4–18.3]) than BD1 (3.74% [2.41–5.51]; c^2^ = 48.7, *p* < 0.0001; Table 1). In addition, the prevalence of RC among BD women (10.9% [8.76–13.4]) was moderately greater (1.51-times) than among men (7.24% [5.20–9.76]; χ^2^ = 5.00, *p* = 0.025; Table 1). The prevalence of RC by diagnosis and sex ranked: BD2 women (16.5% [12.9–20.7]) > BD2 men (13.3% [9.30–18.1]) > BD1 women (5.11% [3.06–7.96]) > BD1 men (2.07% [0.76–4.49]; Table 1).

### Characteristics of RC vs nonRC patients

We compared the 118 RC-BD versus 1143 nonRC-BD patients for differences in a range of demographic, family historical, and clinical factors (Table 2). RC patients were 7.00 years older and been ill for 6.00 more years, but with no significant difference in age-at-onset of BD (29.6 [27.1–32.0] vs. 27.9 [27.2–28.6] years). Data on prospective long-term morbidity were based on samples followed prospectively for ≥ 12 months, for times averaging 11.5 years [9.25–13.7] with RC and 8.81 [8.14–9.48] years with nonRC (*t* = 2.51, *p* = 0.01). Years at risk prior to clinic intake also were greater among RC (15.2 [13.0–17.3]) than nonRC patients (12.4 [11.6–13.0; *t* = 2.30, *p* = 0.02). Patients with RC also were much more likely to be treated with mood-stabilizing agents. RC patients also had selectively higher ratings of cyclothymic temperament and were more likely to report early sexual (but not physical) abuse.

**Table 2:** Factors associated with rapid cycling in bipolar disorder patients

Factor	Measure or proportion [with 95% CI]			χ^2^ or *t*-score	*p*-value
	RC	nonRC	RR		
Subjects (n)	118	1143	1/9.69	–––	–––
RC > nonRC					
Switch with antidepressant (%)	77.5 [68.1–85.1]	35.5 [33.1–38.0]	2.18	70.9	< 0.0001
Inferior treatment response (%)	51.8 [38.0–65.3]	16.1 [13.0–19.7]	3.22	40.5	< 0.0001
Predominant depression	85.0 [76.9–91.2]	58.6 [55.6–61.3]	1.45	28.6	< 0.0001
Total years ill	23.0 [20.5–25.4]	17.5 [16.7–18.3]	1.31	4.04	< 0.0001
Children/person	1.38 [1.09–1.68]	0.90 [0.82–0.98]	1.11	3.49	0.0005
Mood-stabilizer use	84.0 [63.9–95.5]	49.8 [43.5–56.1]	1.69	10.7	0.001
Age at intake	45.0 [41.5–48.5]	39.4 [38.2–40.5]	1.14	3.06	0.002
Ever married (%)	58.8 [49.1–67.9]	44.0 [40.0–47.0]	1.34	9.09	0.003
Siblings/person	4.40 [3.85–4.95]	3.68 [3.53–3.82]	1.20	2.99	0.003
Initial depression (%)	76.2 [67.7–83.5]	63.2 [61.0–65.5]	1.21	8.37	0.004
Follow-up (years)	11.5 [9.25–13.7]	8.81 [8.14–9.48]	1.30	2.51	0.01
Suicidal acts (%)	25.2 [17.7–34.0]	16.7 [15.0–18.5]	1.51	5.72	0.02
Medical comorbidity (%)	73.4 [60.9–83.7]	59.0 [54.9–63.1]	1.24	5.00	0.03
Intake depression (HDRS)	18.5 [13.1–23.8]	12.5 [10.8–14.2]	1.48	2.23	0.03
Cyclothymic temperament	6.78 [5.65–7.92]	5.48 [5.11–5.85]	1.24	2.16	0.03
Early sexual abuse (%)	28.6 [14.6–46.3]	15.6 [18.8–20.0]	1.83	3.80	0.05
nonRC > RC					
Antidepressant use (%)	51.9 [48.5–55.3]	58.5 [56.9–60.0]	1/1.13	12.4	0.0004
Initial [hypo] mania or psychosis (%)	8.20 [4.00–14.6]	18.4 [16.6–20.2]	1/2.24	8.12	0.004
Drug abuse (%)	11.7 [6.39–19.2]	23.0 [23.5–25.7]	1/1.97	7.51	0.006
Cigarettes/day	7.32 [4.80–9.85]	10.8 [9.79–11.8]	1/1.48	2.14	0.03

*RR* = rate ratio for RC/nonRC. Prospective assessments of morbidity are shown in Table 3. Factors that did *not differ* significantly between RC and nonRC subjects included: family history (any psychiatric disorder, mood disorders, BD or suicide, and % of relatives with any disorder), early physical abuse, years of education, unemployment, socioeconomic status, separation or divorce, decade of birth, age at first symptoms, syndromal onset, or treatment), first episode mixed manic-depressive, intake ratings of anxiety (HARS) or functional status (GAF), non-cyclothymic affective temperament ratings, course-type (MDI vs. DMI), alcohol abuse, caffeine consumption, obesity (BMI), metabolic syndrome, serum level of TSH, T3 and T4 thyroid hormones, co-occurring anxiety or attention disorders, seasonal mood-shifts, and hospitalizations/year

### Prospective morbidity

Major differences were found with respect to prospectively assessed morbidity (Table 3). As expected, these differences included 3.21-fold higher average overall recurrence rates with RC-BD, due more to recurrences of major depressive episodes (3.35-fold) than [hypo]manic episodes (3.00-fold). Among RC subjects, the annual rate of depression was 1.72-fold greater than the recurrence rate for [hypo] mania (*t* = 2.96, *p* = 0.002). Proportion (%) of time ill overall, in depression, or particularly in [hypo] mania differed less between RC and nonRC subjects (Table 3). Of note, in years prior to clinic entry rates of recurrence among RC patients (overall [2.25-fold] and for depression [3.69-fold] or [hypo] mania [2.76-fold]) were much greater than among nonRC cases, especially for depression, whereas %-time-ill again did not differ significantly overall or for depression or [hypo] mania (not shown). Interestingly, episodes/year decreased significantly, but selectively for nonRC patients, between years before versus during follow-up at the study sites (from 1.86 [1.59–2.12] to 1.13 [1.01–1.26] for nonRC cases [*t* = 4.87, *p* < 0.0001] and from 5.76 [0.53–11.0] to 2.54 [1.91–3.16] for RC cases [*t* = 0.84, *p* = 0.20]), suggesting greater treatment responsiveness among nonRC cases, and perhaps improved treatment at the mood disorder centers. RC patients also did not differ in rates of hospitalizations per year by history prior to entry to the study centers or during prospective follow-up after clinic entry.

**Table 3:** Morbidity during prospective follow-up of 870 bipolar disorder patients for at least one year

Measure	RC	nonRC	Ratio	*t*-score	*p*-value
Episodes/year					
Episodes	2.44 [1.83–3.06]	0.76 [0.70–0.82]	3.21	12.3	< 0.0001
Depressions	1.55 [1.14–1.95]	0.46 [0.43–0.50]	3.35	12.4	< 0.0001
[Hypo] manias	0.90 [0.61–1.19]	0.30 [0.27–0.33]	3.00	9.10	< 0.0001
%-Time Ill					
Overall	31.5 [26.3–36.7]	21.5 [19.9–23.0]	1.47	3.87	0.0001
Depression	22.4 [18.6–26.2]	15.1 [13.9–16.3]	1.48	3.59	0.0003
[Hypo] mania	9.08 [6.83–11.3]	6.34 [5.65–7.02]	1.43	2.39	0.02

### Additional comparisons

A few factors were found to be *less prevalent or smaller* among RC than nonRC BD patients. In addition to the numbers of RC (118) and nonRC subjects (1143), these differences included *lower* risks of drug abuse and of cigarette smoking with RC (Table 2), with no difference in rates of misuse of alcohol.

Finally, many other factors and measures *did not differ significantly* between RC and nonRC BD patients as reported in footnotes of Table 2. Of note, RC patients did not have a higher risk of violent or fatal suicidal acts than among nonRC cases (6.06% [1.68–14.8] vs 14.5% [11.8–17.7]; χ^2^ = 3.60, *p *= 0.06).

### Cycling rate during prospective follow-up

An important comparison was of average cycling rates observed among previously RC vs. nonRC BD subjects followed prospectively for ≥ 12 months, with exposures of 11.5 (RC) and 8.81 years (nonRC) as already noted. The *mean* prospective recurrence rate was 2.44 [1.83–3.06] episodes/year with RC versus 0.76 [0.70–0.82] among nonRC subjects—highly significantly, 3.21-times higher with RC (Table 3). In addition, in the 10.8 [10.5–11.2] years prior to clinic entry, the overall annual recurrence rate again was 3.73-fold higher among RC patients (5.64 [1.38–9.90] vs 1.51 [1.34–1.68]; *t* = 6.25, *p* < 0.0001). *Median* prospective recurrence rates differed by 3.29-fold: 1.69 [inter-quartile range [IQR] = 2.25] episodes/year with RC versus 0.51 [IQR = 0.83] among nonRC BD subjects.

**Fig. 1:**
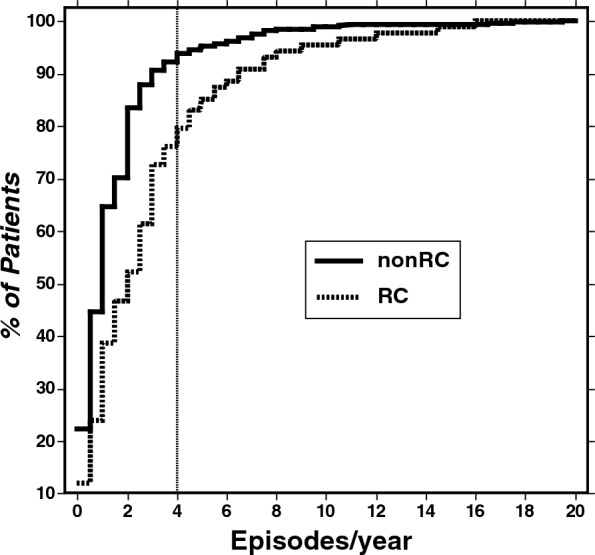
Cumulative histograms of the proportions of previously rapid-cycling (RC; ≥ 4 recurrences in any year) or nonRC BD patients with prospectively ascertained recurrence rates (episodes/year) during follow-up assessments for ≥ 12 months. Prospective recurrence rates were < 4/year among 79.5% [69.6–87.4] of previously RC patients and 93.8% [91.9–95.3] of previously nonRC patients

Analysis of cumulative frequency distributions indicated that average prospective recurrence rates of  ≥ 4/year occurred in only 20.5% [12.6–30.4] of previously RC subjects whereas 79.5% [69.6–87.4] experienced average recurrence rates of  < 4/year. Additional analyses compared BD patients followed for at least one year with rates ranging from 0.50 to 4.00 per year. Most (55%) of previously RC patients experienced prospective recurrence rates of  ≥ 2.0 episodes/year and 30% of previously nonRC patients also cycled at such high rates (Fig. 1), suggesting that RC status is not consistent over time.

We also considered more closely BD patients with versus without prospective recurrence rates of  ≥ 2 episodes/year (more than twice the overall mean rate of 0.84 [0.71–0.92] episodes/year). Factors associated with such elevated recurrence rates were similar to findings with all RC versus nonRC patients (Table 2), with the notable exceptions that associations of higher recurrence rates with BD2 diagnosis and with early sexual abuse were smaller than with all RC/nonRC patients included. Factors strongly associated with recurrence rates of  ≥ 2/year included: mood switching during antidepressant treatment, mean episodes/year (as expected), *less* use of antidepressants, higher cyclothymic temperament ratings, and more suicidal acts. These observations suggest that identified factors may be associated with relatively high mean rates of illness episode recurrences and not highly selectively with RC as defined by a recurrence rate of  ≥ 4.0/year.

### Multivariable modeling of factors associated with RC-BD

Use of logistic multivariable regression modeling for factors associated significantly and independently with RC-BD identified five factors among those identified in preliminary bivariate analyses (Tables 1 and 2). By strength of association they ranked as: [a] an expected higher rate of episodes/year during prospective follow-up, [b] older age at intake, [c] more mood switching during treatment with an antidepressant, [d] diagnosis (BD2 > BD1), and [e] lack of abuse of substances other than alcohol (Table 4).

**Table 4:** Multivariable logistic regression model: factors associated with rapid cycling in bipolar disorder

Factor	OR [95% CI]	χ^2^	*p*-value
More episodes/year	1.21 [1.11–1.31]	21.0	< 0.0001
Older current age	1.03 [1.01–1.05]	13.4	0.0003
More mood switching	2.81 [1.48–5.31]	10.1	0.001
BD2 > BD1	2.32 [1.33–4.02]	8.90	0.003
Lack of substance abuse	3.60 [1.25–10.4]	5.61	0.02

In addition, prospective depressions/year exceeded [hypo] manias/year with RC by 1.72-fold [1.65–1.80], and %-time depressed versus %-time-[hypo] manic by 2.47-fold [2.35–2.60]

## Discussion

This study involved 1261 BD patients (642 BD1, 619 BD2) followed prospectively and systematically for at least one year (average of 11.5 years with RC and 8.81 years with nonRC cases) by the same mood disorder expert (LT). We compared descriptive and clinical characteristics in BD patients with versus without RC, including demographic, family history, clinical factors, and prospective long-term morbidity over at least 12 months. Several noteworthy differences emerged. The observed rate of previous RC in any year among BD patients was 9.36%, which is lower than rates pooled from 12 earlier reports by Carvalho and colleagues [2014], who found a lifetime prevalence of RC in BD of 25.8%–43.0%, and previous-year prevalence of 5.0%–33.3%, with weighted means of 31.5% (lifetime) and 18.1% (previous year) (Carvalho et al. 2014). It might be that in the collection of retrospective data, RC was not recorded consistently or that its risk may have been limited by infrequent use of antidepressants which can facilitate RC (Kukopulos et al. 1980; Tondo et al. 1981; El-Mallakh et al. 2015), as is a standard practice at the study sites. As expected, an RC course with four or more discrete depressive episodes in one year was rare in a separate sample from the same clinics, of 2926 DSM-5-TR unipolar major depressive disorder (MDD) patients (0.76% [0.25–1.76]), at 12.3-times lower risk than with BD.

Higher risk of RC was associated with older age at clinic entry and with a diagnosis of BD2 (15.2%) versus BD1 (3.74%); risk of RC also was highest among women diagnosed with BD2 and lowest among BD1 men (Table 1). These findings are consistent with previous reports in which RC-BD was most prevalent with BD2 among women (Kilzieh and Akiskal 1999; Erol et al. 2015).

The association of older age at presentation with diagnosis of BD2 may reflect a tendency of BD2 patients to seek help later than BD1 whose manic symptoms typically require clinical attention and often lead to hospitalization. BD2 subjects seek treatment for depressive symptoms much more frequently than for hypomanic phases, as they typically do not recognize the significance of hypomanic symptoms and so may experience delayed or overlooked diagnosis and appropriate treatment (Phillips and Kupfer 2013; Baldessarini et al. 2020). Moreover, predominantly depressive polarity, commonly described in RC-BD patients and found in the present study (Table 2), itself has been associated with delay in receiving a correct diagnosis and appropriate treatment (Rosa et al. 2008). Older age of RC-BD also may reflect development of RC later in the course of illness, including with exposure to antidepressant treatment (Kukopulos et al. 1980; Tondo et al. 1981; Azorin et al. 2008; El-Mallakh et al. 2015), perhaps particularly among those with a DMI course, and cyclothymic temperament (Azorin et al. 2008). Antidepressant-exposure also is more likely with BD2 diagnosis, predominant depression, and possibly among women, so as to make cycle-acceleration more likely (Altshuler et al. 1995).

We found higher scores for cyclothymic temperament in RC-BD versus nonRC patients (Table 2). Cyclothymic temperament has been linked to high prevalence of depressive symptoms and mixed states in RC-BD patients (Akiskal et al. 1998; Azorin et al. 2008). Moreover, Koukopoulos and colleagues found that BD patients with cyclothymic temperaments had an early pattern of short, alternating affective oscillations, with an increased risk of developing RC (Koukopoulos et al. 1983). In general, cyclothymic temperament may be considered an indicator of emotional instability.

The higher risk of RC found in this study with BD2 than BD1 is consistent with such a tendency found in a previous meta-analysis (Kupka et al. 2003). Of note, the moderate sex difference found in the present study (Table 1) involves RC among women, rather than a higher proportion of women (typically over-represented in clinical samples) among RC patients, a clarification emphasized in our earlier review of ten studies which did not find a significant effect of sex on risk of RC (Tondo and Baldessarini 1998). Women with BD are more likely than men to develop predominantly depressive polarity (Nivoli et al. 2011; Baldessarini et al. 2012), with increased likelihood of antidepressant exposure (Carvalho et al. 2014). Therefore, the complex relationship between RC course, female sex, and BD2 may, at least in part, reflect predominance of depression in RC-BD with associated use of antidepressants (Coryell et al. 1992; Carvalho et al. 2014). Indeed, our findings suggest that RC categorization was associated with much higher historical and prospective recurrence rates, especially depressive, as well as a lesser excess of %-time ill—that is, with greater RC/nonRC differences in recurrence rates than in %-of-time ill. This pattern may reflect shorter though more frequent illness episodes with RC and tend to limit the expected impact of greater morbidity on functional status.

The prominence of depressive symptoms in RC-BD found in the present study is consistent with previous reports (Coryell et al. 2003; Schneck et al. 2004; Calabrese et al. 2005). Indeed, it has been suggested that highly recurrent depression is a hallmark of RC-BD (Calabrese et al. 2001). Of particular clinical importance, and likely related to the excess of depression with RC, the risk of suicidal acts was significantly, 1.51-times, greater among RC than in nonRC cases (Table 2), in accord with previous similar findings (Coryell et al. 2003; Goldberg et al. 2004; Cruz et al. 2008; Hajek et al. 2008; Garcia-Amador et al. 2009; Undurraga et al. 2012; Valentí et al. 2015). Nevertheless, the present RC-BD patients did not display higher rates of violent or fatal suicidal acts and tended toward lower rates.

Increased suicidal behavior in RC patients may be due to several factors. First, more frequent illness recurrences, especially depressive episodes, even if shorter, may be especially threatening and generate less certainty with discouragement and an adverse effect on morale and confidence in the value of treatment. Second, RC course was associated with cyclothymic affective temperament that may itself contribute to emotional and behavioral instability with increased suicidal risk with higher likelihood of presence of mixed features (Baldessarini et al. 2017; Miola et al. 2021, 2022). Third, abrupt transitions into, and predominance of depression with RC are likely to add to suicidal risk and the presence of mixed features (Coryell et al. 2003). Fourth, use of antidepressants is likely to have further destabilizing effects, including mixed features and agitated depression with additional suicidal risk (Altshuler et al. 1995; Akiskal et al. 2005a, b; El-Mallakh et al. 2015).

The lower indices of morbidity, especially for depression, found during prospective treatment compared to years preceding intake at the study centers may suggest that treatment received at specialized mood disorder centers was especially effective (Schneck et al. 2008). That such improved clinical outcomes were more likely among nonRC patients is consistent with the conclusion that RC patients were less treatment-responsive. The sites involved in the present study follow treatment strategies recommended by Koukopoulos, including avoiding use of antidepressants in RC-BD patients in favor of mood-stabilizers including lithium, lamotrigine, and valproate (Kukopulos et al. 1980). Limited exposure to antidepressants also may explain, at least in part, the lack of increased rates of violent or fatal suicidal acts in the present RC-BD patients and their limited risk of unusually high recurrence rates (Fig. 1).

The present findings also confirm a selective association between early sexual abuse and RC course (Table 2), as had been found in previous investigations (Kupka et al. 2005; Etain et al. 2013; Aas et al. 2014) and appears to be supported by a previous evidence synthesis (Dualibe and Osório 2017). Additionally, we found 1.24-fold higher rates of general medical disorders in RC patients (Table 2), as had been noted previously (Hajek et al. 2008; Kato et al. 2020).

In accord with previous investigations (Nurnberger et al. 1988; Coryell et al. 1992; Lish et al. 1993; Bauer et al. 1994), we did not find greater risk of familial psychiatric illnesses with RC (Table 2). Being married (and consequently more children) was more prevalent among RC patients. Some factors that were notably less prevalent among RC-BD patients, including use of antidepressants as well as abuse of drugs (but not alcohol) and smoking, probably associated with the lesser prevalence of these factors among BD2 (associated with RC-BD) versus BD1 patients (Tondo et al. 2022).

We also found no association of thyroid functioning and RC, though such an association has been proposed (Bauer et al. 1990; Sack et al. 1988) though not always supported (Wehr et al. 1988; Maj et al. 1994; Post et al. 1997). In general, it remains unclear whether hypothyroidism can predispose to RC-BD or whether treatment of BD with thyroid-altering agents such as lithium can contribute to risk of RC (Carvalho et al. 2014; Buoli et al. 2017), although there is support for selective therapeutic benefits of L-thyroxine for RC-BD (Gyulai et al. 2018).

## Limitations

A potential limitation of this study is the retrospective determination of past RC status compared to prospective, long-term assessment of morbidity. However, any such errors are likely to be similar between diagnostic groups and sexes.

## Conclusions

The observed lifetime risk of RC in BD (in any year) was moderate at 9.36%, possibly in part reflecting infrequent use of antidepressants at the study sites. With RC, as expected, average recurrence rates were much higher than among nonRC patients, especially for depression, with much less difference in %-time ill, suggesting shorter episodes. Recurrence rates observed during prospective, long-term follow-up of formerly RC patients were < 4/year in three-quarters and < 2/year among nearly half of them. The risk of greater psychiatric morbidity, of suicidal behavior, and apparent low responsiveness to treatment were important adverse outcomes. Although optimal long-term treatment of RC-BD remains to be clarified, use of antidepressants probably is destabilizing and should be avoided in favor of mood-stabilizers. The apparent lack of greater risk of familial mood disorders with RC may suggest that the condition is often nonfamilial or sporadic in addition usually to representing a transient manifestation of BD.

## Data Availability

Data generated or analyzed during the current study are not publicly available due to confidentiality agreement with study participants but are available from the corresponding author on reasonable request, with confidentiality restrictions.
